# Insights from the field: Evaluating handler feedback on training practices and standards on canine detection testing

**DOI:** 10.1016/j.fsisyn.2026.100717

**Published:** 2026-08-03

**Authors:** Kathleen R. Sucher, A. Celeste Medrano, Adele Quigley-McBride, Paul Bunker, Michelle Karpinsky, Will Chapman, Lauryn E. DeGreeff, Paola A. Prada-Tiedemann

**Affiliations:** a Department of Environmental Toxicology, Texas Tech University, Lubbock, TX, United States; b Department of Psychology, Simon Fraser University, Burnaby, BC, Canada; c Chiron K9, Somerset, TX, United States; d Department of Chemistry and Biochemistry, Global Forensic and Justice Center, Florida International University, Miami, FL, United States; e Department of Forensic Science, Noblis, Reston, VA, United States

**Keywords:** Explosive detection canine (EDC), Canine testing, Canine certification, Standards validation, Canine handler, ANSI/ASB Standard 092

## Abstract

Explosive detection canine (EDC) teams are widely used to find explosives and protect public safety, but training practices and standards vary across agencies/organizations. This study investigated how EDC handlers train their dogs, their familiarity with national standards, and what challenges they face in preparing for effective operation. Data from a nationwide survey of handlers (102 unique responses, 62 complete), across 29 states, and in-depth interviews were conducted during two field evaluations of the ANSI/ASB Standard 092 *Standard for Training and Certification of Canine Detection of Explosives*. Results revealed most handlers train regularly and value certification; however, training structures differ widely. Many handlers reported limited familiarity with national standards and scientific research, and some lacked access to formal training guides, realistic scenarios, or certain explosive materials needed for effective practice. Handlers emphasized the importance of practical training that reflects real-world conditions, including complex environments, low target prevalence, and presence of distractors. They also reported challenges related to limited access to true training materials, uncertainty about how odors change or how canines generalize between like odors, time and resource constraints, and differences between testing formats and field work. Concurrently, many viewed structured evaluations as helpful for identifying weaknesses and improving performance. Overall, the findings suggest successful detection depends on multiple factors working together: consistent standards, access to resources, realistic training, scientific guidance, and a strong handler-canine partnership. Improving communication between researchers, standards organizations, and practitioners, as well as increasing outreach and access to evidence-based information, may help strengthen training, reduce errors, and improve operational readiness.

## 1 Introduction

Canines have extraordinary olfactory and behavioral capabilities and, when partnered with an effective, experienced person (i.e., handler), canine-handler teams provide valuable support in law enforcement, military and private‐contractor settings. For example, the capabilities of canine-handler teams are leveraged by a variety of agencies for screening and locating explosives hidden in cargo, vehicles, baggage, building structures, or on-person screening. Canine-handler teams perform important tasks in real‐world contexts that humans alone could not [1].

Although canine detection teams are widely used, the way that these programs are organized and how teams are trained varies considerably. In law enforcement (LE) contexts, canine-handler teams are often embedded within police or federal units and are typically subject to formal certification and periodic recertification requirements. In contrast, private‐contractor teams (e.g., those working in corporate security or civilian-run forensic contracting) may adopt different training regimens and operational protocols. These differences raise important questions about training consistency, operational readiness, and team coordination across contexts.

In response to these concerns, the Organization of Scientific Area Committees (OSAC) for Forensic Science, Dogs and Sensors Subcommittee developed the ANSI/ASB Standard 092, *Standard for Training and Certification of Detection of Explosives*. This document provides baseline for training, certification, and maintenance practices for explosives detection canine-handler teams, including recommendations related to handler and canine training, assessment procedures, training aid management, and documentation. Although such standards provide a more consistent structure for training and assessment, they do not address how handlers understand, implement, and respond to these recommendations in practice.

Hence, there is a current gap in understanding handlers’ perspectives on training and assessment practices—particularly regarding what information handlers find useful, where uncertainty arises, and what challenges they encounter when attempting to implement standards in operational settings. The current paper addresses this gap by presenting quantitative survey data alongside qualitative insights from interviews with canine handlers conducted during a proof-of-concept black-box study assessing the OSAC explosives detection standard (results reported in Karpinsky et al., 2025) [2]. Together, these data provide insight into current training and assessment practices, associated challenges and resource needs, and factors that may influence operational readiness in explosives detection contexts.

### 1.1 The importance of standardized training and assessment approaches

There are large discrepancies in training methodologies, even within canine‐detection programs. Some agencies and jurisdictions make use of guidelines that have a robust scientific foundation, but many training paradigms rely on experience-driven or tradition-based approaches. At present, there is limited empirical evidence examining how variation in training practices affects operational performance outcomes, particularly in explosives detection contexts [[3], [4], [5]]. However, the available literature suggests that training conditions can meaningfully influence canine performance in operational settings. For example, when canine-handler teams are expected to operate under double-blind conditions, they are likely to benefit from being trained under those same conditions [6].

Initiatives such as OSAC, run by the National Institute of Standards and Technology (NIST), aim to encourage consistency between and within disciplines and programs by providing a fixed standard for canine detection training and assessment [2,7]. At the same time, the operational environment for detection dogs and their handlers has become increasingly complex, thus effective standards in training and assessment must also reflect these evolving security threats [2,6]. The growing diversity of homemade explosives and improvised hazardous materials require dogs to detect a wider array of odorants, many of which can vary in formulation and can be masked by a variety of distractor odors. Research on odor generalization shows that dogs trained to detect one preparation of a target substance may not always reliably detect modified or unconventional versions that they may encounter in the field [[8], [9], [10], [11], [12], [13], [14]].

To address these challenges, training regimens must systematically vary the complexity and level of odor presented to promote discrimination, generalization, and performance under more realistic conditions [2,7]. Common approaches include the incorporation of varied environmental training scenarios, diverse training aids, and the use of single- and double-blind search designs, which increase task difficulty and better approximate real-world conditions [6,[10], [11], [12]]. While these elements improve the operational relevance of training and assessments, clear and standardized guidance regarding the appropriate training practices would help address these emerging challenges in canine detection and ensure that, regardless of the particular canine-detection team on duty during an incident, the teams are ready to detect these real-world hazards.

### 1.2 Ensuring the readiness of the handler for operational contexts

Although canines serve as the primary detection tool in operational searches, handlers do not merely “operate” the dog. Instead, canine detection functions as a coupled human–animal system in which the handler acts as a cognitive partner who interprets canine behavior, manages search strategies, and adapts dynamically to operational environments [15]. Effective detection work therefore depends not only on the technical proficiency of the canine, but also on the handler's situational awareness, interpretation skills, and decision-making abilities. As Gillespie (2023) notes, handlers must develop these skills in tandem with the dog's individual differences in personality, behavior, and operational performance [5].

Because handlers play such an important role in interpreting canine behavior, deficiencies in training, experience, or access to evidence-based guidance may reduce detection reliability and increase variability in performance [[16], [17], [18]]. Human factors research demonstrates that handler cognition, expectations, and prior beliefs can influence search outcomes by biasing alert interpretation and contributing to false positives [[15], [16], [17],^19^,^20^]. These findings highlight the importance of structured training approaches that reduce expectancy effects and strengthen objective decision-making under uncertainty, including the incorporation of single- and double-blind search designs into training and assessment protocols [6,15,21,22].

Ensuring handler readiness for operational contexts therefore requires ongoing development that extends beyond initial certification. Handlers benefit from deliberate practice in varied and unpredictable environments that mirror field conditions, including low-prevalence target scenarios, complex search areas, and evolving odor presentations. Access to flexible training environments, scientifically grounded guidance, standardized certifications, and regular performance feedback may help strengthen handler confidence, improve interpretation skills, and support reflective practice. Standardized training and evaluation structures may also help reduce variability in preparation across agencies by establishing clearer expectations regarding operational readiness and performance [2,7].

In addition, the quality of the handler–canine partnership remains a critical determinant of operational success. Effective working relationships are built through repeated interaction, mutual trust, and shared experience under realistic conditions rather than through informal or purely companion-based dynamics [23]. Research suggests that stable, well-practiced teams demonstrate greater accuracy, lower stress behaviors, and improved resilience in complex environments [6,23]. Accordingly, training and evaluation structures should account not only for technical proficiency, but also for the development and maintenance of effective handler–canine coordination and communication.

### 1.3 Bridging the gap between research and practitioners

Despite growing research on canine detection performance, many handlers may lack access to current scientific findings, evidence-based guidance, or standardized training and assessment procedures. Gaps remain in the systematic documentation and synthesis of handler perspectives regarding training practices, odor generalization, training-aid accessibility, operational realism, and the implementation of standards, despite growing literature addressing technical aspects of canine training aids and odor calibration [24,25]. These issues are important because handler beliefs, expectations, and training experiences can directly influence detection outcomes [6].

At the same time, even when standards exist, how they are implemented in practice may vary substantially across agencies and organizations. A systematic review of canine olfactory detection research highlighted considerable methodological variability within the field and noted that this inconsistency complicates the translation of laboratory findings into operational practice [2]. In some cases, programs described as using “standard” training procedures continue to rely heavily on legacy or tradition-based methods rather than approaches that have been formally validated. Such variability arises from differences in available resources, training aids, and access to operationally realistic training environments. OSAC has therefore identified the development and validation of more robust standards as an ongoing research priority [2].

Accordingly, standardized assessments should be evaluated not only empirically, but also through feedback from the handlers and trainers expected to implement them in practice. The current study addresses this issue by examining handler experiences during a validation study of ANSI/ASB Standard 092, a standard developed by the OSAC Dogs and Sensors Subcommittee. Specifically, this paper evaluates handler feedback regarding training and assessment practices, standards awareness, odor-related science, access to training aids and realistic training environments, and the operational relevance of assessment procedures. By examining the experiences and perspectives of operational handlers, this study aims to identify practical, and human-factors challenges that may inform future standards development, training practices, and operational readiness in canine detection contexts.

## 2 Training, certification, and standard knowledge survey

### 2.1 Method

The first aim of the study was to conduct a survey (Florida International University Institutional Review Board Protocol #23-0572) of the explosive-detection canine community in the United States inquiring about the knowledge and implementation of OSAC National Registry Standard 092, *Standard for Training and Certification of Canine Detection of Explosives* (ANSI/ASB Std 092).

The survey was created and administered using Qualtrics survey software and distributed via social media platforms, such as Facebook and LinkedIn, as well as targeted emails to canine detection associations. Limitations associated with this form of recruitment include selection bias for known organizations and potentially under sampling handlers who are not active on social media. Participating was voluntary and responses were anonymous. Participants did not need to respond to all questions but were encouraged to do so. There were 102 total unique responses received, with 62 canine handlers completing the survey (though not necessarily providing a response to every question). The survey had two sections—the first focused on canine training practices, training aids, and certification information. The second section assessed participants’ prior knowledge of and engagement with OSAC and SWGDOG (Scientific Working Group on Dog and Orthogonal Detector Guidelines). The SWGDOG, established in 2004, developed foundational documents and best-practice guidance for detector dog teams, including recommendations for training, certification, and operational use. In 2014, its role transitioned to the Organization of Scientific Area Committees for Forensic Science Dogs and Sensors Subcommittee, which now develops consensus-based standards for canine detection disciplines. Each OSAC standard incorporates core components including terms and definitions; canine team initial training requirements (handler, dog, and team); canine team assessments; certification requirements; maintenance training; training aid storage and handling; and canine team records and document management.

### 2.2 Results and discussion

**Fig. 1:**
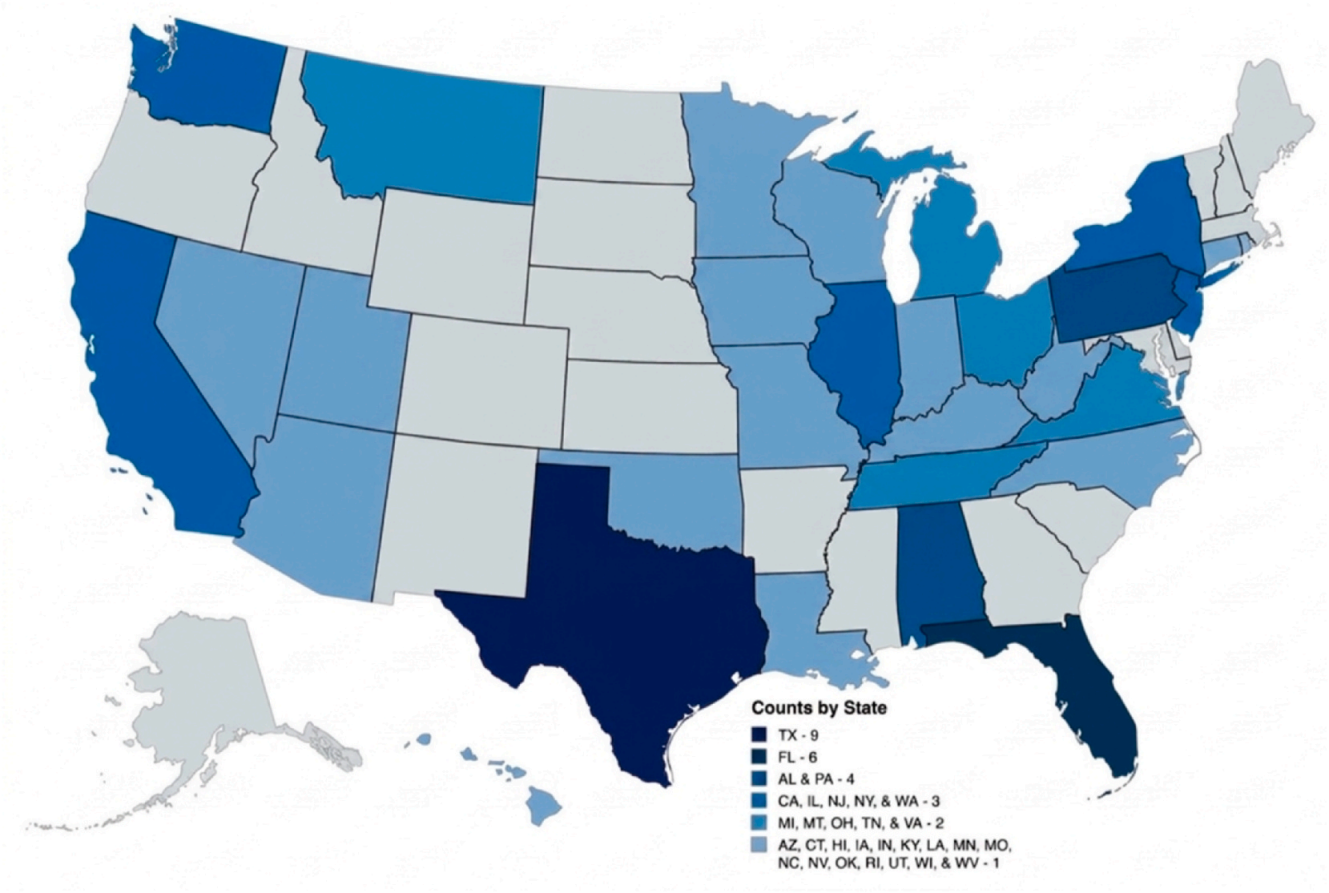
Distribution of responses by location in the United States (darker blue indicates a larger number of responses; gray indicates no responses).

Respondents were from 29 different states (*n* = 62 responded), with the largest representation from Texas (*n* = 9) and Florida (*n* = 6). Fig. 1 depicts the state representations, while Table 1 provides a summary of respondents’ training and certification practices. Some respondents reported that they are currently working with more than one explosive detection canine (EDC). In those cases, data reported in this study represent only the data from the first EDC. Most participating EDC handlers reported training more than 4 h per week (90%), while the remaining reported training less than 4 h per week, suggesting that regular training is a priority across most agencies and practitioners. The training protocols used were both formal and informal, with 31% of respondents reporting the use of training guidelines developed by a national agency or certifying body. Another 31% reported relying on guidance developed by their own department, and the final 26% indicated no use of formal training guidelines. A similar distribution of responses was received when respondents indicated the guides and frameworks used to train their EDC. Thus, while time spent training varies very little, the structure and source of training guidance is much more diverse.

**Table 1:** Descriptive statistics on respondents’ EDCs currently in training or operational.

Variable	Response	% of total
Amount of training	>4 h p/w <4 h p/w	90% 10%
Sources of Training Guides	A National Agency Their own Department or Agency No training guide Other sources	31% 31% 26% 12%
Training guides used to train your 1st EDC?	National Agency Local Agency Own Dept/Agency None	31% 11% 31% 26%
Certification required	Yes – required No – voluntary	92% 8%
Certification Status	Certified with a National Agency Not Certified with a National Agency	74% 26%
What certification does your 1st EDC have?	National Agency Local Agency Own Dept/Agency None	74% 16% 16% 6%

Almost all respondents (92%) reported that certification was required rather than voluntary, and most (74%) indicated that their EDC held national-level (e.g. NAPWDA, IPWDA) certification. A smaller proportion hold local (16%) or department-level certifications (16%), and very few (6%) indicated no current certification. These findings suggest that certification is common in this domain and that national-level certification is the most frequently obtained type among respondents.

Table 2 summarizes respondents’ awareness and adoption of (SWGDOG. In general, familiarity with this organization was limited. Over half of respondents reported no familiarity with the organization (58%) or its standards (69%). Similarly, 60% were not aware of the SWGDOG SC8 Substance Detector Dogs, Explosive Detection from 2007 [26]. Most (77%) indicated their EDC had not been certified under the standard though some respondents reported planning to use all of the standard or portions of the standard in the future.

**Table 2:** Descriptive statistics regarding EDC handler awareness of SWGDOG Standards.

Variable	Response	% of total
Are you familiar with the SWGDOG organization?	No Yes – Somewhat Yes – Very	58% 24% 18%
Are you familiar with any of the standards put forth by the SWGDOG organization?	No Yes – Somewhat Yes – Very	69% 16% 15%
Are you aware of SWGDOG SC8 - *Substance Detector Dogs, Explosive Detection* (2007)?	No Yes, I have heard of it. Yes, I have read it. Yes, I have read it and commented on the standard during the open comment period. Yes, I was involved in writing the standard.	60% 13% 15% 0% 2%
Have you trained your EDC(s) according to SWGDOG SC8?	No No, but I plan to implement all or portions in my training. Yes	50% 28% 22%
Has your EDC been certified according to SWGDOG SC8?	No No, but I plan to certify using this standard when possible. Yes	77% 10% 13%

Table 3 presents data concerning knowledge and implementation of standards developed by the OSAC Dogs and Sensors Subcommittee, as well as respondents’ broader familiarity with the OSAC and SWGDOG organizations. Similar to responses regarding SWGDOG, most survey respondents reported that they were not familiar with the OSAC Dogs and Sensors Subcommittee (69%) or its standards (65%). Nevertheless, respondents demonstrated greater awareness of ANSI/ASB Standard 092 (2021), with 34% reporting that they had at least heard of the standard and 21% indicating that they had read it, compared to only 30% familiarity with the SWGDOG SC8 Substance Detector Dogs Explosive Detection Standard (2007), despite the latter having been publicly available for a substantially longer period. Overall, respondents were also more familiar with the work of the SWGDOG organization (42%) than with the OSAC Dogs and Sensors Subcommittee (30%), which is unsurprising given that SWGDOG existed earlier and operated for a longer period than the OSAC subcommittee. However, fewer respondents indicated that their EDC had been formally certified (14%) or trained (23%) according to ANSI/ASB Standard 092, although some participants reported plans to implement the standard, or portions of it, in the future for training (34%) and certification (26%).

**Table 3:** Descriptive statistics regarding EDC handler awareness of OSAC and ANSI/ASB Standard 092.

Variable	Response	% of total
Are you familiar with the OSAC Dogs and Sensors Subcommittee?	No Yes – somewhat Yes – Very	69% 19% 11%
Are you familiar with any of the standards put forth by the Dogs and Sensors Subcommittee?	No Yes – Somewhat Yes – Very	65% 22% 13%
Are you aware of ANSI/ASB Standard 092: *Standard for Training and Certification of Canine Detection of Explosives*?	No Yes, I have heard of it. Yes, I have read it. Yes, I have read it and commented on the standard during the open comment period.	44% 34% 16% 5%
Have you trained your EDC(s) according to ANSI/ASB Standard 092?	No No, but I plan to implement all or portions in my training. Yes	37% 34% 23%
Has your EDC been certified according to ANSI/ASB Standard 092?	No No, but I plan to certify using this standard when possible. Yes	46% 26% 14%

**Fig. 2:**
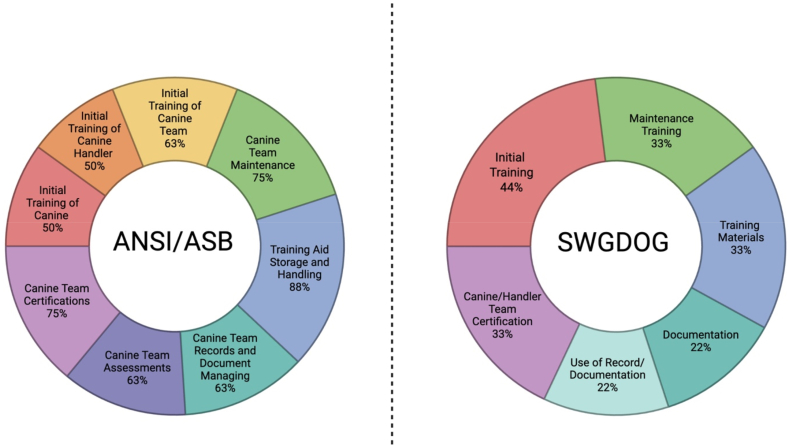
Percent survey respondents using sections of the ANSI/ASB or SWGDOG standards.

Respondents were asked to identify which sections of each document they incorporate into their protocols (Fig. 2). The most widely referenced section of Standard 092 among handlers was the one on training aid storage and handling, with 88% of respondents reporting that they use this section. Sections addressing canine team maintenance and certification were also widely used among survey respondents (75%). For the SWGDOG document, the section on initial training was most popular (44%). Other sections of this document covering maintenance training, training materials, and certification also appeared to be commonly used guidance materials (33% each). Though a promising start, the entire document should be used by the canine detection community to promote consistent and standardized training of EDCs across the country.

Overall, these results suggest that, while certification itself is a common practice, awareness and full adoption of specific national standards documents varies considerably among EDC handlers. The lack of overall familiarity may be addressed through outreach and open communication between these organizations and the general public. By increasing practitioner knowledge about the standards that are available through outreach events, such as seminars or workshops, this will foster the implementation of this standard into the routine training and certification of canine-handler teams and, ideally, lead to agencies and organizations adopting these protocols too.

## 3 Interviews: on-site handler feedback

### 3.1 Method

This study assessed canine-handler team performance on assessments outlined in Standard 092 and compared standard assessment performance to success in additional scenarios designed to mimic operational deployments. The study was held in three different U.S. sites with a total of 56 teams (law enforcement, government and private organizations), though the interviews were only conducted during the trials at sites #2 and #3. Further study details can be found in Karpinsky et al. (2025) [2]. Karpinsky and co-authors evaluated the operational performance of explosive detection canine (EDC) teams by comparing standardized certification testing with realistic field scenarios. Using a single-blind design, handlers were unaware of explosive placements while researchers measured detection accuracy, false alerts, and missed detections during both ANSI/ASB Standard 092 certification searches and operationally realistic scenarios.

The certification assessments included vehicle, room, parcel, baggage, and odor recognition tests (ORTs) conducted under standardized conditions. The ORTs used 24-box lineups containing explosive odors, blanks, and distractor odors to isolate basic odor recognition ability while minimizing environmental complexity. Operational scenarios introduced more realistic challenges, including people screening, box piles, and outdoor searches with competing non-target distractions. The study used the six explosive odor categories required under ANSI/ASB Standard 092. Results showed substantial variability among teams and across explosive odor types. ORT findings demonstrated that performance deficits were present even under simplified conditions, suggesting that some detection failures were related to odor recognition training rather than environmental factors alone. Results depicted that performance during standardized certification assessments generally paralleled performance in operational scenarios. The authors reported variability in canine performance and discussed inconsistencies in training methods and training aids as potential contributing factors, emphasizing the need for standardized validation and training practices for explosive detection teams.

The handler interviews were conducted during the trials and were semi-structured in nature, with a list of planned questions (the full list can be found in Supplementary Materials), and leeway to follow conversations as they developed. Handlers were interviewed and engaged in conversation during the trial day while they waited between assessments. Thus, at times they were asked about their general experience training an explosives detection canine and working in the field detecting explosives, and other times they were specifically asked to comment on their experience completing the Standard 092 certification tasks.

The interviews were recorded by the interviewer and then transcribed using Sonix (https://sonix.ai), an AI transcription software. Once complete, the AI generated transcriptions were reviewed and edited by research assistants to ensure accuracy, as well as redacted to ensure the information provided was de-identified. Once the transcriptions were available, the interviewer read through all transcriptions and extracted sections of the transcriptions that were relevant to the questions the interviews were designed to address. This reduced set of transcriptions was then analyzed to extract broad themes or issues raised by the handlers.

To ensure that the content themes extracted by the interviewer were not subject to confirmation or hindsight biases, two independent research assistants with no knowledge of the study, canine-handler team dynamics, or the interviewer's hypotheses were trained to extract themes from these same transcriptions. They were instructed to read through the full set of redacted transcriptions twice before going through a third time and noting emerging themes. The fourth read through was used to refine and write descriptions of the themes. Finally, they were asked to use the fifth read through to choose quotes that best exemplified the themes they identified.

Once the research assistants were finished extracting their themes independently, they met to discuss the themes they identified and reconcile them (e.g., decide when two of their themes were substantively the same and combine them under an agreed upon label and description). This resulted in a final list of themes with associated quotes for which they both agreed were prominently featured in the interviews. The themes generated independently by the two research assistants and those generated by the interviewer were compared by determining whether the themes were conceptually equivalent in meaning and scope, or if some of the themes represented examples of broader categories identified by the research assistants or the interviewer. Themes were considered convergent when they represented the same underlying construct, even if labeled differently, or could reasonably be subsumed into a larger construct. Discrepancies were also resolved via discussion between the research assistants and the interviewer to determine whether differences reflected distinct concepts or semantic variation in labeling only. Where appropriate, the final list of themes includes some that were merged or retained as subthemes if consensus among those on the team was reached after a discussion.

Given the small number of themes and the absence of a predetermined coding scheme applied to turn qualitative data into categorical or quantitative data, convergence was assessed qualitatively rather than through a quantitative inter-rater reliability statistic. The independently-generated theme lists are provided in Supplementary Materials to allow readers to evaluate the degree of overlap directly. The final list of themes and subthemes is discussed below.

### 3.2 Results and discussion

The interviews provided additional context for the findings from the Black-Box Study (Karpinsky et al., 2025) [2] and the survey results presented here. In particular, they offered insight into how handlers understand and implement training and assessment practices, the challenges they face in accessing research-based guidance and preparing for operational contexts, and the constraints that shape their practices. A qualitative analysis of the interview data revealed several recurring themes related to variability and uncertainty in training practices, challenges in recreating realistic operational conditions, resource and structural constraints, and the role of handler interpretation in canine detection performance.

**Fig. 3:**
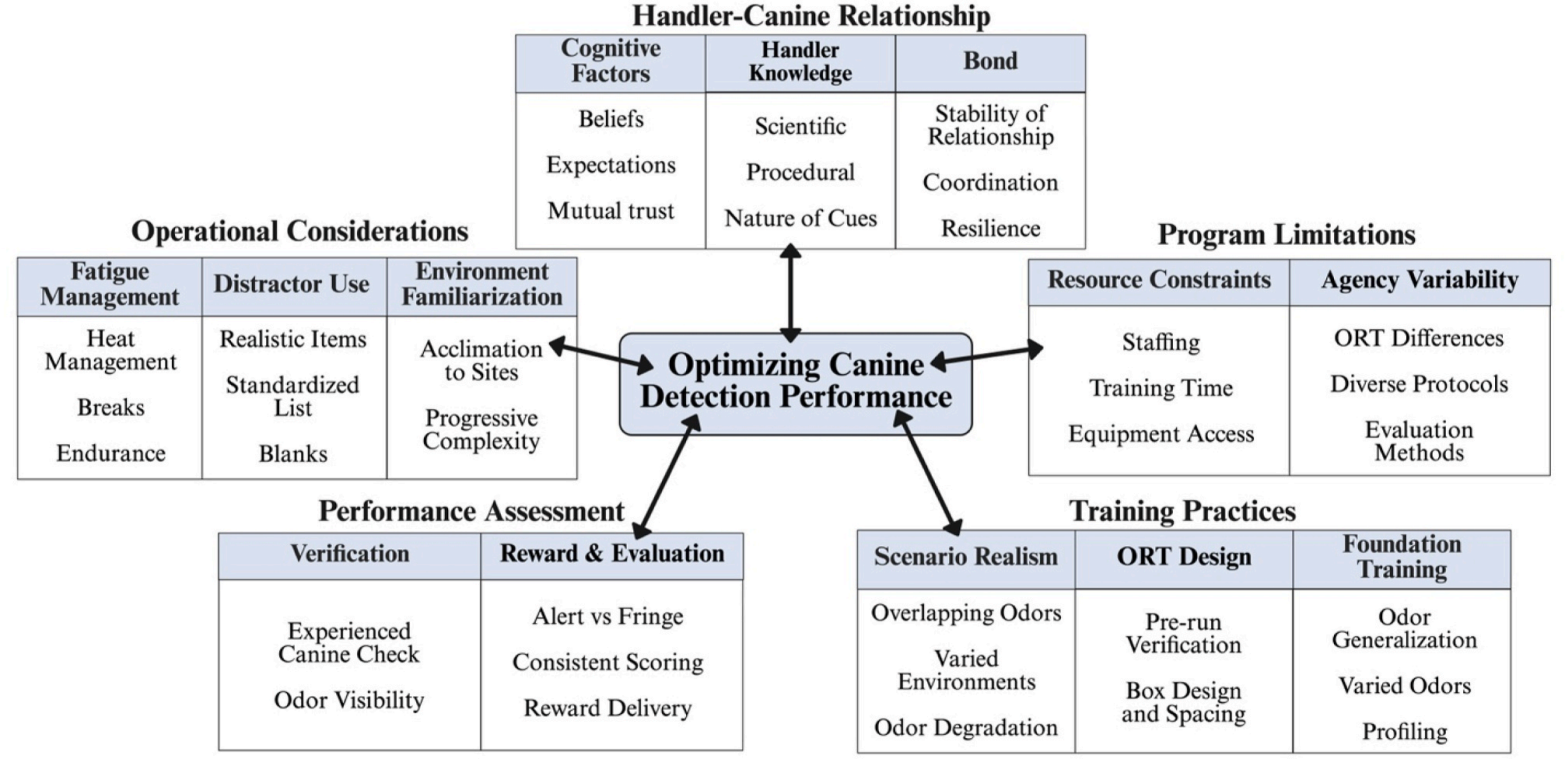
Themes extracted from the handler survey, in combination with previous literature, on optimizing canine detection performance.

Together, these themes highlight how training, assessment, and operational performance are shaped by the interaction between canine capabilities, handler decision-making, and the environments in which teams operate. Fig. 3 illustrates how these themes map onto broader factors that interact to influence canine detection performance.

#### 3.2.1 Performance assessment

One purpose of the interviews was to get feedback on the handlers’ experiences participating in the Standard 092 certification trial as a form of performance assessment. The reported handler feedback reflects the standard implementation used in this study rather than deficiencies of Standard 092. We summarize here key feedback that may help improve the implementation of this standard within the canine detection community.

##### 3.2.1.1 Odor recognition test

Many recommendations on improving the standard revolved around the ORT, particularly the type of containers used for the test. An odor recognition test in canine detection evaluates a dog's ability to accurately identify a trained target odor while ignoring non-target odors and environmental distractions. The test typically consists of a controlled search involving multiple containers in either a line-up or carousel style design, in which one or more hides contain the target odor, and the remaining hides serve as blanks and distractors. The dog is expected to independently locate the target odor and perform its trained alert behavior under single/double blind conditions to minimize handler influence and ensure objective assessment of odor recognition accuracy [7]. Standard 092 intentionally provides flexibility in assessment design, and implementation details may vary between agencies while remaining compliant with the standard. In the current study, cardboard boxes were used in a line up design, which were placed 3 feet apart. Within the implementation used in this study, several handlers suggested using containers that were affixed to the ground or weighted to reduce movement during searches. These comments reflected participant experiences with the assessment configuration used in this study rather than criticism of Standard 092, which allows flexibility in assessment design. Handlers also noted that the box arrangement made it difficult to maneuver through the search area and keep track of which boxes had already been cleared. Several handlers said that it was “*… a better setup to do the ORT first, and then that gives you like a sense if your dog's struggling generally that day*.” Several handlers viewed the ORT used in this assessment as a useful indicator of canine readiness before proceeding to more complex assessment scenarios.

Several handlers, particularly those from government agencies with greater access to space and resources, discussed standardized approaches to odor recognition testing used in their own routine training and certification. These handlers reported that their ORTs often involve a small number of containers positioned at substantial distances from one another—sometimes in entirely different rooms—to enable discrete, binary “yes/no” decisions with minimal spatial or odor overlap. Consequently, some handlers felt the ORT differed substantially from the testing environments they were accustomed to, which they believed affected performance. Most other handlers reported fewer concerns about search area size or spacing, emphasizing instead the physical stability of the containers.

Because multiple canine teams work the same scenarios during assessments, handlers were concerned about contamination—prior teams might leave traces throughout the scenario, making it difficult for their own canine to focus on the target odor(s) [17]. They recommended multiple rooms set up in a similar fashion, thus minimizing the number of canines going through the same room. Although this may be difficult due to facility limitations, handlers viewed it as an important consideration for future assessments. The problem of restricted access to a diverse range of training aids for foundational odor learning (discussed further later in 3.2.6) was also mentioned in reference to ORT performance which meant some canine-handler teams struggled to train robust associations between targets and rewards [24,27].

##### 3.2.1.2 Distractors and blanks

Several handlers from agencies that prioritized highly controlled testing environments noted that the range of distractors used and the proximity of intentionally placed distractor odors to target odors during the trial differed from their typical training and certification practices. However, operational environments rarely involve isolated target odors, and handlers noted that overlapping or competing odors are common in real-world searches [2,6,[28], [29], [30]]. Handlers acknowledged that these aspects of the assessment reflected operationally realistic search conditions, although several noted that similar scenarios were not routinely incorporated into their agency's training. Other handlers did not object to the placement of distractors but instead noted their struggles with blank or distractor items that were novel to their canines. Some handlers thought their dogs were alerting to novel or unfamiliar stimuli because, in this type of context, they had learned that anything unusual was likely to result in a reward. One handler explained, “*She alerted on TADD … she's never seen TADD … to her that's something foreign*.”

Several handlers mentioned that a list of distractors that might be assessed would be useful for them during training. One handler indicated that the list should differentiate between “negative controls” (items used to package explosives e.g., gloves and tape) and “distractors” (non-target stimuli placed within a search area e.g., humans, food, and animal odor). Some handlers suggested that “negative controls” were the most difficult to train for because they are “*part of the scent picture*.” Although providing a detailed list of distractors and negative controls could help handlers prepare for assessments, doing so may also reduce operational realism of training and assessment. Additionally, this would mean that canines are proofed (trained) to ignore specific odors rather than mirroring a realistic assessment for distraction. Maintenance training should therefore incorporate varied and challenging distractor exposures to reinforce reliable odor discrimination and performance during certification and deployment.

The concerns raised by handlers regarding distractor placement and evaluation criteria reflect broader issues related to standardization, construct validity, and operational relevance in canine detection testing. Highly controlled testing formats may not adequately represent operational environments where odor plumes overlap and competing background odors are common [7,19]. When training and certification protocols emphasize isolated targets, dogs may be less prepared to interpret overlapping or unusual odor presentations, potentially increasing fringe behaviors or partial responses for novel distractors. Together, these observations highlight the importance of standardized training guidance and operationally realistic assessment structures.

##### 3.2.1.3 Feedback on evaluators and reward opportunities during assessment

Handlers expressed differing views regarding evaluator error calls, the timing of feedback during trials, and the distinction between a true alert and a fringe response. Several handlers suggested that these issues might be mitigated by clearly outlining what to expect for error calls and feedback before beginning the trials so that the handlers can plan how to respond and reward their canine during the sessions. These mixed responses highlight the difficulty of creating a standardized practice that is applicable and well-suited for all canine teams. However, listening to those in the relevant community and carefully considering their feedback is a crucial first step in developing standards that accommodate as many views and approaches as possible.

##### 3.2.1.4 Benefits of the standard 092 assessment trial

Relatedly, the current study was often described as revealing weaknesses and unanticipated deficiencies in their training programs. Many handlers viewed this as particularly valuable when framed as a training opportunity rather than solely a certification exercise. One handler said that, in general, “… *their dogs did not run perfectly … and that's what I need more of. Like, I need to* [mess] *up here, not out there*.” Handlers viewed exposure to more difficult scenarios than those typically encountered in training or certification as highly valuable, especially for private contractors who struggle to set up scenarios like that with the resources they have available to them. Handlers also highlighted the value of deliberately challenging scenarios with one participant suggesting, “… *we expect this … we want to test the threshold of the dog …*” and build their confidence in the field. That said, many also suggested that handlers would need to train with the Standard 092 assessment in mind as many would struggle given that many of the scenarios were very different than what they were used to.

##### 3.2.1.5 Challenges associated with standardization

In addition, handlers noted substantial variability in training standards and practices across organizations, highlighting the challenges of achieving alignment among multiple agencies and organizations in the pursuit of standardization. One handler felt that “*There will never be a standard that everyone agrees on … every canine unit's function is different.*” The concerns raised by handlers in this study, together with prior findings in the literature, [19], indicate that some flexibility within standardized approaches may be necessary to accommodate variation in agency roles, operational demands, and available resources. Standards that are too strict could be rejected by certain agencies if they are logistically problematic within their institutional context. Participant-handlers also highlighted limited oversight regarding their performance and maintenance of skills after certification. Once a canine-handler team is certified, there is no requirement that they continue training and improving. One handler pointed out that, “*You guys make these cert standards … but then there is no requirement for teams to train …*” which means that teams may not prioritize maintenance and improvement-focused training post-certification. Although maintenance training is included within current OSAC standards—which recommend a minimum of 16 h of monthly maintenance training for operational teams—handlers nevertheless expressed concern about variability in how ongoing training requirements are implemented and monitored across organizations.

#### 3.2.2 Handler-canine relationship

Handlers emphasized that canine-handler teams differ fundamentally from forensic tools that are expected to produce consistent outputs regardless of the operator. As one handler suggested, although canines are technically “tools,” training approaches cannot be identical across canine-handler teams, and the same canine may not interact with a different handler in the same manner [2,6,18]. They explained, “*you can take* [a type of firearm] *and take it from me … hand it to him … it's going to fire the same … The dog's not the same way. I used to take my dog and give it to another handler. You [know] how the dog works? Yes. Will it work? Give 100%? No, because it requires that emotional connection*.”

Handlers consistently emphasized that detection outcomes are a team effort that depends on coordination and trust between the handler and their dog. Success in training, testing, or operational contexts depends not only on the readiness of the canine and the canine's behavior, but also on how those behaviors are interpreted and reinforced by the handler. Canine responses may be subtle or ambiguous, requiring handlers to make judgment calls based on their experience with that particular canine and the surrounding context. Handlers therefore viewed training as important not only for developing canine performance, but also for strengthening communication, coordination, and mutual trust within the team.

Many handlers explained that training was as much for them as it was for the canine because it helped them learn how to coordinate with, interpret, and trust their canine's responses. One handler explained, “*I was always taught you trained for the alert, but then you read the change … so* [the question is] … *are you going to be able to read that* [correctly]*?*” Handlers also noted that their own behavior and emotional state could unintentionally influence canine performance. As one participant described: “… *if you go in there and … get frustrated … the dog now becomes frustrated because he can sense that you're frustrated.*” Together, these observations suggest that standardized approaches to training and assessment should account for natural variation in how handlers and canines communicate and work together, and ordinary variations in the dynamic between the handler and the canine day-to-day. Standards that incorporate shared performance goals while allowing flexibility in training strategies may therefore be more practical and more widely accepted across agencies and operational contexts [18].

Interviews also revealed areas where handlers expressed uncertainty or beliefs about odor behavior that may not fully align with the current scientific literature. For example, some handlers discussed odor movement, odor degradation, or “stale” odors in ways that reflected inconsistent or incomplete access to research findings. These beliefs may influence training decisions and operational expectations, particularly when handlers are attempting to generalize from personal experience in the absence of accessible scientific guidance. Importantly, these observations do not reflect a lack of engagement or interest among handlers. Rather, they highlight the need for clearer communication of relevant scientific findings in formats that are accessible and useful to practitioners working in operational settings.

#### 3.2.3 Operational considerations

There is a clear link between canine welfare and performance in both assessments and operational contexts, so many handlers felt that additional breaks should be incorporated into assessments. Breaks allow dogs to rest and “reset,” particularly in hot environments, and may help reduce fatigue [2,23,31]. Conversely, several handlers noted that the assessments were relatively short compared to operational deployments and therefore did not adequately test canine stamina or endurance. These differing perspectives suggest that the ideal assessment structure may depend on the specific goals of the assessment and the operational contexts for which teams are being prepared. Many handlers also believed that canine performance improved on the second day of testing because the dogs had acclimated to the environment. However, handlers emphasized that operational deployments often occur in unfamiliar locations where acclimation opportunities may be limited. Consequently, many viewed the ability to perform reliably across both familiar and unfamiliar environments as an important operational skill.

Many participants expressed concern that routine training practices were overly simplified or predictable, and that this formed the basis for the skills and expertise of many canine-handler teams that are operational. For example, one handler said, “*A lot of people are just like … ‘let me just put a pound in a drawer and then we're just going to run it’ …*” Handlers worried that reliance on relatively straightforward scenarios may lead some teams to overestimate their operational readiness and struggle in more complex environments, including assessments such as Standard 092. Simplified training scenarios may also reduce opportunities for dogs to rely primarily on olfaction and for handlers to practice interpreting subtle behavioral changes [6]. These considerations are supported by the results of the trial associated with these interviews—canine-handler teams who performed well on the more challenging scenarios designed to mimic operational contexts were more likely perform well on the Standard 092 assessment [3].

Another dominant theme across interviews was the difficulty of replicating real-world operational conditions during training, particularly in explosives detection contexts. One handler stated: “*There are times where we are running like 500 cars … they're all blank … it's really hard to create … a training scenario …* [that mirrors these conditions]”. Another explained: “*I work the festivals. Terrible because what … what I had learned, and especially with bomb work, we don't have … a lot of hits …*” In operational explosives detection work, target odors are encountered relatively infrequently. However, the consequences of failing to detect an explosive are extremely high [7]. One handler described this pressure directly: “[If they find narcotics or not], *somebody gets paid … get it back next time … if you miss a bomb, that's it. Everybody dies.*”

These operational realities create unique training challenges because handlers must maintain canine vigilance despite long sequences of blank searches [2,7,15]. Low target odor frequency may increase the risk of fatigue, performance degradation, and handler bias if these factors are not explicitly addressed during training through approaches such as variable reinforcement schedules [6,27,31]. Accordingly, teams should be trained and assessed on their ability to reliably clear blank scenarios while maintaining sensitivity to target odors.

#### 3.2.4 Training practices

Handlers frequently described canine detection outcomes as being shaped by learning processes that can both support and undermine performance. Many emphasized the importance of ensuring that canines are learning the intended associations and are reinforced only for appropriate behavioral responses. Small issues during training, they explained, can unintentionally teach canines incorrect associations. One handler noted: “*Your trainer actually becomes part of the dog's scent picture … you need to make sure … you put gloves on … or you'll set your own dog off.*”

Reward structures during training were also identified as critical for preparing teams for operational work. One handler explained: “*You … could have alerted … you rewarded them. But then when we take away the reward, we're still technically …* [in the scenario and needing to continue working].” Many handlers expressed interest in research on canine cognition, reinforcement schedules, and learning processes that could help optimize training practices and improve operational performance (for examples of such research, see Refs. [6,[31], [32], [33], [34]]).

The other main areas where handlers expressed both interest and uncertainty involved odor concentration, odor movement, and odor generalization—that is, whether a canine trained on one version of an odor will reliably detect related variants encountered in operational settings. These concerns are important because operational scent pictures often differ substantially from training aids due to contamination, degradation, packaging, environmental exposure, or variation in formulation.

Research suggests that reliable odor generalization depends on several factors, including the chemical similarity of odorants, the specific volatile compounds used during training, and individual differences between canines [10,11,34,35]. Consequently, training on a single example of a target odor may not reliably prepare a canine to detect chemically related or altered variants encountered in the field [10,11,34]. Previous research also supports training with varied odor quantities, sources, and contexts to promote more stable detection performance and reduce contextual learning effects [11,12,24,28]. One handler illustrated this concern by discussing chlorates, perchlorates, and consumer fireworks. They noted that dogs trained directly on fireworks reliably detected fireworks, whereas dogs trained only on chemically related compounds sometimes failed to alert to fireworks in operational contexts. This example reflects broader concerns among handlers about whether training approaches adequately prepare canines for real-world odor variability.

Handlers consistently identified structural, systemic, and resource-related barriers that limited their ability to implement ideal training practices. Even handlers working in government or law enforcement agencies indicated that it could be difficult to prioritize training and allocate the resources necessary for high-quality assessments when agencies were stretched thin. These concerns were often linked to staffing shortages and limited time for maintenance training: “*I can count on two hands the times I've been able to run my dog … because we're so short staffed …*” Many handlers therefore described the trial as a rare opportunity to identify weaknesses in a controlled setting, prompting reflection on current practices and discussions with supervisors about modifying training strategies. Importantly, handlers generally did not view imperfect performance during the assessment as failure. Instead, they emphasized the value of structured training environments in which teams can safely make mistakes, learn from them, and improve without operational consequences.

#### 3.2.5 Program limitations

Although handlers generally supported standardized training and assessment approaches, many described disconnects between standards, research, and real-world practice. Several handlers indicated that “… *they only knew about OSAC because of the trial …*” meaning participation in the study represented their first meaningful exposure to OSAC and its role in canine detection standards. While some participants had heard of organizations such as the North American Police Work Dog Association (NAPWDA) and the Scientific Working Group on Dog and Orthogonal Detector Guidelines (SWGDOG), understanding of the roles and purposes of these organizations was often limited. The trial therefore also served as an introduction to the broader landscape of standards, oversight, and validation efforts within canine detection.

Despite limited familiarity with formal standards, many handlers demonstrated a clear understanding of why standardized and validated assessments are important. Participants frequently emphasized that assessments should evaluate not only basic odor recognition skills, but also the ability to apply those skills in realistic operational contexts. Several described layered testing systems already used within highly resourced agencies. For example, one handler explained that the Department of Defense (DoD) conducts a “*basic run of the mill certification*” followed by more realistic validation exercises every six months in which teams must apply their skills in “*some type of real tactical situation*”. These observations suggest that many handlers value assessment structures that extend beyond simple certification and incorporate operationally realistic performance testing.

Although many handlers lacked familiarity with the scientific literature underlying canine detection standards, they consistently expressed interest in accessible, research-based training guidance and in expanding their awareness of work conducted in government and academic laboratories that may not be readily integrated into their operational context. Their focus was less on specific scientific questions than on how empirical research can be translated into practical training approaches that improve operational preparation and readiness. Thus, handlers expressed strong support for the efforts of our research team (“… *there has to be some level of validation … they're going to go … out in some type of real tactical situation …* [and need to know they are capable of succeeding in that task].”) Participants were generally supportive of the efforts of the research team and viewed empirically validated approaches as potentially valuable for improving operational readiness. Importantly, when the assessment revealed areas where performance could improve, handlers were often eager to learn how to address those weaknesses.

At the same time, participants described difficulty accessing or applying relevant research findings in operational settings. This challenge was reflected both in direct comments from handlers and in discussions about odor behavior, odor movement, and odor generalization that did not always align with the current scientific literature. One private contractor emphasized the urgency of these issues, explaining that “… [information about odor generalization and odor movement in operational settings is] *real-world stuff we need to know now … this is not stuff we need to know in six months*.”

A consistent concern across interviews was that standards often specify what teams should achieve while providing limited guidance on how to achieve those outcomes in practice. This issue is closely related to the structural, systemic, and resource-related constraints affecting training [6]—Without a clear understanding of how to operationalize the performance expectations and certification requirements outlined in assessments such as Standard 092, many teams may struggle to adapt their training practices and may therefore resist changes to certification procedures. Standard 092 already provides structured guidance across key components, including handler, canine, and team-level training and certification requirements; however, handlers emphasized that greater awareness and more consistent application of these components are needed to ensure the standard is used effectively in practice. They suggested that clearly articulated training expectations, when understood and actively implemented within organizations, could help handlers and trainers better align training practices with organizational needs and advocate for additional resources and training opportunities. In this way, more effective use of existing standards such as Standard 092 would better position handlers to use the standard as a practical tool to justify requests for additional training support.

Finally, many handlers—particularly those working outside large government agencies—identified access to appropriate training aids as one of the greatest barriers to preparing canine-handler teams for operational deployment. Private contractors, in particular, described difficulty obtaining both training aids and training environments that allowed them to run operationally realistic scenarios: “*If you are private, you're never going to have access to that stuff …*” Several handlers argued that if certification standards require teams to demonstrate detection of particular explosives, then those materials, or validated alternatives, must also be available for training purposes. These concerns are especially important because developing effective training aids is scientifically challenging. Ideally, a training aid should produce an odor profile that closely matches the true material. However, for many explosives, researchers do not yet fully understand which odor components are most relevant to canine perception [36].

Handlers also expressed concerns about commercially available alternative training aids, with some preferring true materials because they were uncertain whether alternative aids accurately represented operational odors. These concerns are supported by research showing that some alternative aids may not fully replicate the odor signatures of true explosive materials [36] [22,[37], [38], [39]]. One underlying challenge is that the chemicals most easily detected using laboratory instruments are not necessarily the same chemicals most salient to a canine [37,40]. As a result, validating training aids requires not only chemical analysis, but also behavioral testing with canines themselves to determine whether the aids produce appropriate detection responses [36,37,41]. Together, these concerns reinforce the need for scientifically validated training aids and clearer guidance about how such aids should be incorporated into realistic training scenarios.

## 4 Conclusion

This study aimed to address a current gap in understanding canine handlers’ perspectives on training, assessment, and operational readiness using both quantitative survey data and qualitative interview data. Survey responses from explosive detection canine handlers across the United States indicated substantial variability in training structures, sources of guidance, and familiarity with national standards documents, despite relatively consistent reports regarding time devoted to training. Although certification was common among respondents, awareness and adoption of organizations and standards such as OSAC and SWGDOG remained limited, highlighting the need for greater outreach and communication between standards organizations and the canine-handler teams these efforts are intended to support.

The qualitative interviews provided additional insight into how handlers interpret and implement training and assessment practices, the challenges associated with translating training into operational contexts, and the structural constraints that shape these processes. Across interviews, handlers consistently emphasized the importance of operational realism, scientifically grounded training practices, and accessible guidance that can be implemented within the practical limitations of their working environments. Participants also highlighted substantial barriers to implementing ideal training practices, including limited access to training aids, staffing and time constraints, difficulty recreating operational conditions, and uncertainty regarding odor behavior and odor generalization. These barriers appeared particularly pronounced for private contractors and teams operating outside large government agencies.

At the same time, handlers generally viewed the Standard 092 assessment positively and described it as a valuable opportunity to identify weaknesses in training programs that might not otherwise become apparent during routine exercises. Many emphasized that challenging and operationally realistic assessments may improve readiness by exposing canine-handler teams to conditions that more closely resemble real-world deployments. However, handlers also stressed that implementing more rigorous standards successfully depends on whether teams have access to the resources, facilities, training opportunities, and evidence-based guidance necessary to meet those expectations in practice.

Taken together, these findings suggest that improving canine detection performance requires more than the development of well-defined standards and assessment procedures. Effective implementation also depends on understanding how canine-handler teams learn, train, and operate within real-world environments and organizational constraints. The findings further highlight the importance of translating empirical research related to odor chemistry, canine cognition, reinforcement, and operational performance into forms that are accessible and useful to practitioners. By integrating survey findings, handler interviews, and operational assessment experiences, the current study provides insight into the practical, cognitive, and systemic factors that shape explosives detection performance and offers guidance for developing more consistent, evidence-based canine detection practices moving forward.

## Funding

The author(s) declare that financial support was received for the research and/or publication of this article. Funding for the project was provided by the 10.13039/100000161National Institute of Standards and Technology from the grant 2021-NIST-MSE-01.

## CRediT authorship contribution statement

**Kathleen R. Sucher:** Data curation, Formal analysis, Investigation, Writing – original draft, Writing – review & editing. **A. Celeste Medrano:** Data curation, Formal analysis, Investigation, Writing – original draft, Writing – review & editing. **Adele Quigley-McBride:** Conceptualization, Data curation, Formal analysis, Investigation, Methodology, Supervision, Writing – original draft, Writing – review & editing. **Paul Bunker:** Investigation, Methodology, Writing – review & editing. **Michelle Karpinsky:** Investigation, Methodology, Writing – review & editing. **Will Chapman:** Investigation, Methodology, Writing – review & editing. **Lauryn E. DeGreeff:** Conceptualization, Funding acquisition, Methodology, Project administration, Resources, Supervision, Writing – review & editing. **Paola A. Prada-Tiedemann:** Conceptualization, Funding acquisition, Methodology, Project administration, Resources, Supervision, Writing – original draft, Writing – review & editing.

## Declaration of competing interest

The authors declare that they have no known competing financial interests or personal relationships that could have appeared to influence the work reported in this paper.
