# Supplementary material for: Insights from the field: Evaluating handler feedback on training practices and standards on canine detection testing
Source: Forensic Sci Int Synerg. 2026 Aug 3;13:100717. doi: 10.1016/j.fsisyn.2026.100717 (PMC13470439; doi:10.1016/j.fsisyn.2026.100717)
Supplement: Supplementary file 1 [file mmc1.docx]

**Table S1**
*Thematic Alignment Across Independent Coders and Final Consensus Themes.*

| **Domain** | **Research Assistant 1** | **Research Assistant 2** | **Consensus Theme Description** |
| --- | --- | --- | --- |
| Handler Influence & Interpretation | Lazy approaches to training among some trainers/handlers  Realistic training is difficult | Handler can influence the canine and their training in lots of ways | Handler can influence detection outcomes, including interpretive reinforcement of ambiguous alerts |
| Canine Learning & Behavior | Clever dogs try to find shortcuts | Knowing when to reinforce dogs during training | Avoiding shortcut behaviors |
|  | Reward Confusion |  | Clarity and consistency in rewarding |
|  | Training to handle novelty | Uncertainty about what odors to train dogs to detect and avoid | Avoid alerts to novel/unfamiliar odors; unintended scent associations |
|  | Unintended scent pictures |  | Wide variability in odor presentation affects canine performance |
| Training & Certification Quality/ Ecological Validity | Methodological validation in training | How best to approach training structure and conditions | Need for rigorous, ecologically valid training and assessment practices |
|  | Realistic training is difficult | Access to training aids and other training resources is difficult | Barriers to implementing realistic training scenarios and limited access to training materials |
| Structural & Systemic Issues | Training and certification inconsistencies | Inconsistent training standards | Variation in training standards and protocols across units, as well as limited oversight |
|  |  | Limited post-certification oversight and guidance. |  |
|  | Methodological validation in training | How best to approach training structure and conditions | Need for empirically validated training structures |

***Note.*** Research assistants independently reviewed and coded interview transcripts prior to meeting for consensus discussion. Initial themes were generated inductively by each coder without collaboration. During the consensus meeting, overlapping themes were merged, discrepancies were resolved through discussion, and theme labels were refined for conceptual clarity.

**Table S2**
*Thematic Alignment Across Consensus Themes from Independent Coders and Themes Developed by the Interviewer.*

| **Consensus Theme Description** | **Interviewers Theme Descriptions** |
| --- | --- |
| Handler can influence detection outcomes, including interpretive reinforcement of ambiguous alerts | Challenges associated with nerves and novel assessment scenarios |
| Avoiding shortcut behaviors | Canines try to learn the most efficient route to a reward |
| Clarity and consistency in rewarding | Concerns over rewarding during training or assessments when they do not know where the alerts were |
| Avoid alerts to novel/unfamiliar odors; unintended scent associations | What distractors and controls to use |
| Wide variability in odor presentation affects canine performance | What odors to train with, as well as amounts, set up, surrounding circumstances |
| Need for rigorous, ecologically valid training and assessment practices | Need for and interest in robust, standardized training strategies based in research/science |
| Barriers to implementing realistic training scenarios and limited access to training materials | Access to training aids is limited, especially for private contractors/civilians |
| Variation in training standards and protocols across units, as well as limited oversight | Limited knowledge of OSAC and standard development/exposure to standards |
| Need for empirically validated training structures | Limited knowledge of existing canine detection research and how to access this research |

**Table S3**

*Summary of Themes and Representative Handler Quotes*

| **Theme** | **Representative Quote(s)** |
| --- | --- |
| Performance Assessment | “For most of the handlers, they only knew about OSAC because of the trial… they had only heard of other K9 specific organizations…”  “…their dogs did not run perfectly… and that’s what I need more of. Like, I need to [mess] up here, not out there.”  “…there has to be some level of validation… the certification is your basic run of the mill certification… but then they do the validation where… they’re going to go put it out in some type of real tactical situation…”  “There will never be a standard that everyone agrees on… every canine unit’s function is different.” |
| Handler-Canine Relationship | “I was always taught you trained for the alert, but then you read the change… so… are you going to be able to read that? That’s what I want to know…”  “…if you go in there and… you get frustrated… the dog now becomes frustrated because he can sense that you're frustrated.”  “The dog’s not the same way… it requires that emotional connection.” |
| Training Practices | “Your trainer actually becomes part of the dog’s scent picture… you need to make sure… you put gloves on… or you’ll set your own dog off.”  “You… could have alerted… you rewarded them. But then when we take away the reward, we're still technically like… [in the scenario]…”  “…some dogs that train on RDX will then generalize, but some dogs won’t…”  “It’s part of the scent picture… you use it enough that it…”  “She alerted on TADD… she’s never seen TADD… to her that’s something foreign.” |
| Operational Considerations | “There are times where we are running like 500 cars… they’re all blank… it’s really hard to create… a training scenario…”  “A lot of people are just like… ‘let me just put a pound in a drawer and then we're just going to run it’…”  “…they want us… to get aggravated and frustrated… we expect this… we want to test the threshold of the dog.” |
| Program Limitations | “If you are private you’re never going to have access to that stuff…”  “I can count on two hands the times I’ve been able to run my dog… because we’re so short staffed…”  “You guys make these cert standards… but then there is no requirement for teams to train…”  “…[information about odor generalization and odor movement in operational settings is] real-world stuff we need to know now… this is not stuff we need to know in six months.” |
